# Establishment of hypertension risk nomograms based on physical fitness parameters for men and women: a cross-sectional study

**DOI:** 10.3389/fcvm.2023.1152240

**Published:** 2023-09-12

**Authors:** Yining Xu, Zhiyong Shi, Dong Sun, Goran Munivrana, Minjun Liang, Bíró István, Zsolt Radak, Julien S. Baker, Yaodong Gu

**Affiliations:** ^1^ Faculty of Sports Science, Ningbo University, Ningbo, China; ^2^ Faculty of Kinesiology, University of Split, Split, Croatia; ^3^ Faculty of Engineering, University of Szeged, Szeged, Hungary; ^4^ Research Institute of Sport Science, University of Physical Education, Budapest, Hungary; ^5^ Department of Sport and Physical Education, Hong Kong Baptist University, Kowloon, Hong Kong SAR, China

**Keywords:** hypertension, nomogram, risk factor, predictive model, LASSO, physical fitness

## Abstract

**Objective:**

This study aims to establish hypertension risk nomograms for Chinese male and female adults, respectively.

**Method:**

A series of questionnaire surveys, physical assessments, and biochemical indicator tests were performed on 18,367 adult participants in China. The optimization of variable selection was conducted by running cyclic coordinate descent with 10-fold cross-validation through the least absolute shrinkage and selection operator (LASSO) regression. The nomograms were built by including the predictors selected through multivariable logistic regression. Calibration plots, receiver operating characteristic curves (ROC), decision curve analysis (DCA), clinical impact curves (CIC), and net reduction curve plots (NRC) were used to validate the models.

**Results:**

Out of a total of 18 variables, 5 predictors—namely age, body mass index, waistline, hipline, and resting heart rate—were identified for the hypertension risk predictive model for men with an area under the ROC of 0.693 in the training set and 0.707 in the validation set. Seven predictors—namely age, body mass index, body weight, cardiovascular disease history, waistline, resting heart rate, and daily activity level—were identified for the hypertension risk predictive model for women with an area under the ROC of 0.720 in the training set and 0.748 in the validation set. The nomograms for both men and women were externally well-validated.

**Conclusion:**

Gender differences may induce heterogeneity in hypertension risk prediction between men and women. Besides basic demographic and anthropometric parameters, information related to the functional status of the cardiovascular system and physical activity appears to be necessary.

## 1. Introduction

As one of the most prevalent risk factors for cardiovascular diseases in recent decades, hypertension has been shown to have a high probability of causing serious health damage. A substantial body of evidence suggests that hypertension is one of the primary causes of cardiovascular diseases and premature death worldwide (1, 2). Since 2000, various risk prediction models have been established to assess the risk of hypertension incidence in different populations. For instance, a study published in 2019 used Logistic regression analysis to estimate the association between cardiovascular risk factors and hypertension. It demonstrated that physical activity, dietary habits, body mass index, and the presence of diabetes and chronic kidney disease are potent risk factors for hypertension (3). Furthermore, in 2018, a research team reported that, after multivariable adjustments, the risk factors for developing hypertension included age, gender, body mass index, blood pressure, and serum uric acid. They emphasized that high serum uric acid was a significant predictor of hypertension prevalence (4).

However, these risk prediction models for hypertension have their limitations. In developing these models, earlier studies employed single-factor analyses to validate the models' predictive power, which was a multivariate issue, or used stepwise regression to derive results, leading to model overfitting (5, 6). Additionally, many primary predictors in previous models relied on biochemical parameters from laboratory tests, which demand high-quality testing equipment and skilled personnel.

In recent decades, parameters from sports medicine and physical fitness perspectives, such as handgrip strength, forced vital capacity, and performance of exercise assessment, has gained attention in medical care and public health (7–9). These parameters are simpler to collect and have fewer requirements for devices and personnel compared to medical laboratories. However, the predictive power of these new parameters remains ambiguous and is often debated, as most of these parameters have been validated and derived using single-factor analyses or stepwise regression. Moreover, significant gender differences in physical fitness between men and women can affect the discrimination, specificity, and sensitivity of these parameters, complicating consensus in clinical practice (10–12).

This study meticulously crafted hypertension risk nomograms for Chinese male and female adults, adhering to the Transparent Reporting of a Multivariable Prediction Model for Individual Prognosis or Diagnosis (TRIPOD) statement. Beyond basic demographic and anthropometric parameters, the research incorporated intricate facets such as biochemistry, daily dietary habits, sleep patterns, and physical activity, ensuring a comprehensive predictor selection process. The sophistication of the least absolute shrinkage and selection operator (LASSO) regression analysis was employed, adeptly addressing the challenges of multicollinearity inherent in numerous potential variables laden with confounding factors. This rigorous approach augments result accuracy. Participants were judiciously segregated into distinct cohorts for external validation of the model's precision and stability. All pertinent diagnoses were predicated upon immediate blood sampling and meticulous laboratory testing, eschewing potential reporting biases.

## 2. Methods

### 2.1. Data source

The study was designed as a cross-sectional study. All potential indicators of hypertension were identified by screening the medical records system of the physical examination center in the Research Academy of Grand Health, Ningbo University, Ningbo, China, from September 2020 to August 2022.

### 2.2. Participants

The data were obtained from adults who participated in the annual routine health examinations at the physical examination center. The inclusion criteria for participation in this study were as follows: (1) Over 18 years old. (2) Absence of any musculoskeletal disabilities or clinical exercise contraindications. (3) Stable residency in Ningbo for at least 6 months or permanent residency. (4) No swelling, inflammation, severe pain, recent hand injuries, or hand surgeries in the past 6 months that would prevent grip strength testing. (5) Absence of respiratory and lung-related diseases or other reasons that would prevent completion of the forced vital capacity testing in the last 6 months.

The exclusion criteria for this study were: (1) Presence of cognitive impairment and/or inability to participate. (2) Presence of musculoskeletal disabilities and/or clinical exercise contraindications. (3) Clinical diagnosis of severe organic diseases, such as tumors or having undergone major surgeries. (4) Data that deviated more than two standard deviations from the mean, was identified as an outlier. This threshold, widely accepted in statistical analyses, ensures data integrity and reduces the influence of extreme values. (5) Missing data due to the nature of the data collection from the grouped annual routine health examinations. This includes participants who, either by choice or oversight, did not undergo specific tests such as forced vital capacity (FVC) or grip strength and those who did not provide blood samples for reasons such as vasovagal reactions.

### 2.3. Outcome

The outcome of this study was the incidence of hypertension and its odds ratio. Hypertension was defined according to the 2022 version of the Chinese Clinical Practice Guidelines of Hypertension (13) as having a systolic blood pressure (SBP) ≥ 130 mmHg and/or diastolic blood pressure (DBP) ≥ 80 mmHg. Participants who reported daily use of antihypertensive drugs were also considered hypertensive. The assessment of the outcome to be predicted was not conducted blindly.

### 2.4. Predictors

18 potential predictors from demographic, anthropometric, biochemical, daily life, and physical fitness perspectives were collected. The measurement procedures for data collection included: (1) Researchers confirmed the time and venue with participants at the physical examination center 7 days before the start of the physical examination, enabling them to notify the participants and recruit them to the study; (2) Each participant was asked to fast for at least 10 h before the examination, with examination and screening starting at 8 a.m.; (3) First, a physical examination was conducted to collect data on body weight (kg), height (cm), chest circumference (cm), hipline (cm), waistline (cm), systolic blood pressure (SBP, mmHg), diastolic blood pressure (DBP, mmHg), and resting heart rate (beats/min); (4) Fasting venous blood sampling; (5) The participants were provided with breakfast after fasting venous blood sampling. This was provided at the physical examination center with 250 kcal calories from carbohydrates, 150 kcal calories from protein, and 100 kcal calories from fat; (6) After breakfast, the participants were asked to complete a questionnaire survey according to the instructions given by medical staff. The contents related to clinical indicators were completed by specialists; (7) 1 h after breakfast, the grip strength and forced vital capacity of the participants were assessed; (8) 2 h following breakfast, postprandial venous blood sampling.

Blood pressure was meticulously gauged using an advanced automatic electronic blood pressure monitor (HBP-9021, Omron Corp., Kyoto, Japan). For each assessment, three consecutive measurements were taken, and their average was judiciously computed to represent the definitive blood pressure value for that particular session. Body mass index (BMI) was calculated by dividing the body weight (kg) by the square of body height (m).

The fasting and postprandial blood samples were analyzed to obtain essential biochemical variables, including fasting blood glucose (FBG), postprandial blood glucose (PBG), plasma total cholesterol (TC), triglyceride (TG), low-density lipoprotein-cholesterol (LDL-C), and high-density lipoprotein-cholesterol (HDL-C). All blood samples were collected through standardized processes and stored under standard conditions (refrigeration at 4°C) before being sent to the laboratory (within 2 h of collection). An automatically calibrated biochemical analyzer was used for the biochemical analysis (AU5800 Clinical Chemistry Analyzer; Beckman Coulter Inc., Brea, CA, USA).

The questionnaire survey included questions about basic demographic information and conditions of the daily diet, sleep, and physical activities. The questionnaire was administered in paper format, with staff assisting each participant on a one-on-one basis to answer all the questions.

Forced vital capacity testing followed standardization for spirometry as outlined previously (14). Grip strength was measured using standardized protocols: (1) Participants were asked to hold a Smedley spring-gauge hand-held dynamometer and to squeeze the handle as hard as they could for 2 s with the value recorded in kilograms by the researcher before the device was reset; (2) Participants were instructed to stand without arm support but were allowed to conduct the test with arm support and seated if required; (3) The test was performed up to 6 times, 3 times in each hand, alternating between hands; (4) The maximum grip strength (kg) measured was recorded.

The definition of hyperlipidemia followed the international classification, diagnostic, and therapeutic perspectives of hyperlipidemias, while the definition of hyperglycemia followed the recent international clinical and medical consensus on the diagnosis of hyperglycemia, prediabetes, and diabetes (15–18). The diagnosis criteria of hypertension, hyperlipidemia, and hyperglycemia are provided in Table 1. Participants were ascertained to possess a cardiovascular disease (CVD) history if they had been diagnosed with either hyperlipidemia or hyperglycemia, or if they consistently reported the daily administration of medications pertinent to these conditions. This delineation aligned with established clinical paradigms that emphasized the significance of a comprehensive cardiovascular history, encompassing not only overt cardiac events but also related metabolic conditions and therapeutic interventions (19).

**Table 1:** The diagnosis criteria of hypertension, hyperlipidemia, and hyperglycemia.

Items	Diagnosis criteria
Hypertension	• SBP ≥ 130 mmHg • and/or DBP ≥ 80 mmHg
Hyperlipidemia^a^	• TC > 5.17 mmol/L (200 mg/dl) • and/or TG > 2.3 mmol/L (200 mg/dl)
Hyperglycemia	Including diabetes and prediabetes Diabetes: • FPG ≥ 7.0 mmol/L • and/or PBG ≥ 11.1 mmol/L Prediabetes: • FPG from 6.1 mmol/L to 7.0 mmol/L • and/or PBG from 7.8 mmol/L to 11.1 mmol/L

SBP, systolic blood pressure; DBP, diastolic blood pressure; TC, plasma total cholesterol; TG, plasma total triglyceride; FPG, fasting blood glucose; PBG, postprandial blood glucose.

^a^ No international uniform clinical or medical standard.

The data collection and data analyses for the study were approved by the Research Academy of Grand Health, Ningbo University. All experimental procedures were conducted following international guidelines and regulations by trained researchers. All researchers involved in data collection had completed professional medical training protocols. The study protocol was approved by the Ethics Committee of the Research Academy of Grand Health, Ningbo University (No. 20190098) and conducted following the Declaration of Helsinki and subsequent amendments (20). All patients provided signed written informed consent forms. Ethical permission and a sample of informed consent are provided in the Supplementary Files.

### 2.5. Sample size

The determination of the requisite sample size was based on the prevalence of hypertension specific to China (13), the number of potential variables (21), and the $R_{CS}^{2}$ (22).

The $R_{CS}^{2}$ was carefully selected as a conservative value, representing the expected model performance, as defined by the Cox-Snell R-squared statistic. The anticipated value of $R_{CS}^{2}$ was of paramount importance, representing the ratio of signal to noise. This ratio profoundly influenced the estimation of multiple parameters and the potential susceptibility to overfitting. In scenarios where the signal-to-noise ratio was expected to be high (with $R_{CS}^{2}$ approaching 1 for a prediction model), identifying genuine patterns became easier, reducing concerns of overfitting and allowing the estimation of more predictor parameters. Conversely, when this ratio was expected to be low (with $R_{CS}^{2}$ nearing 0), the challenge of distinguishing true patterns increased, raising the potential for overfitting and limiting the reliable estimation of predictor parameters. Thus, $R_{CS}^{2}$ essentially mirrored the coefficient of determination R2, which quantified the proportion of outcome variance explained by the prediction model, consistently ranging between 0 and 1 (21).

Given that the outcome measure was the diagnostic determination of hypertension, a binary variable, it was essential to ensure that the sample size was large enough to approximate the overall outcome proportion with adequate precision, as shown in the subsequent equation:

(1) $$n={\left(\frac{1.96}{0.05}\right)}^{2}\phi (1-\phi )$$

*Φ*, representing the anticipated outcome proportion within the study population, was initially assumed to align with a prevalence of 41% for hypertension. However, in light of further considerations and to ensure regional specificity, *Φ* was adjusted to reflect the prevalence of hypertension specific to China, which stands at 27.9% (13). With this adjustment, the value of *n* was determined when *Φ* equated to 27.9% (0.279), resulting in a value of 309. It is worth noting that the model operates under the assumption of a 27.9% prevalence with a 5% absolute precision to ensure the highest reliability of the findings.

In this study, with the number of potential variables being 18, notably less than 30, it was crucial to select a metric that would accurately reflect our model's prediction precision across all predicted values. The mean absolute prediction error (MAPE) emerged as an ideal choice in this context. MAPE measures the average error in the model's estimated outcome probability for new individuals from the target population. This metric is particularly relevant for binary logistic prediction models, as it offers a direct measure of the average prediction error, ensuring that our model's predictions are both precise and consistent. Although the literature generally recommends a MAPE of 0.050, a stricter threshold of 0.040 was adopted in this study, emphasizing our dedication to achieving optimal prediction accuracy (23). Hence, the sample size should conform to the following equation:

(2) $$n=\exp\left(\frac{-0.508+0.259\ln(\phi )+0.504\ln(P)-\ln(\mathrm{MAPE})}{0.544}\right)$$

When *P* represents the number of potential variables, which is 18 in this study, the sample size is determined when *Φ* equals 27.9% (0.279), resulting in a value of 977.

Finally, based on Riley's study, the $R_{CS}^{2}$ should be at least 0.2 for logistic regression, and the model's shrinkage factor (S) should be no less than 0.9 and could be articulated as:

(3) $$S=\frac{R_{CS}^{2}}{R_{CS}^{2}+\delta_{\max}(R_{CS}^{2})}$$

(4) $$n=\frac{P}{\left(S-1)\ln(1-(R_{CS}^{2}/S)\right)}$$

The *δ*_max_ was recommended to be 0.05, at which point, the $R_{CS}^{2}$ value for hypertension ranged from 0.245 to 0.485 (24). Consequently, the sample size was 1,271. However, when S equated to 0.9, the minimum sample size was 567.

In conclusion, based on the calculations derived from the aforementioned equations, the maximum value was used to ensure that the sample size for this study exceeds 1,271. This decision was made to guarantee a sufficiently robust sample size, thereby ensuring the reliability and predictive capability of our model.

### 2.6. Statistical analysis

Participants with missing or incorrect data were excluded from the statistical analysis in line with the study's exclusion criteria. The R software (version 4.1.2; R Foundation for Statistical Computing, Vienna, Austria) was used for the statistical analysis. The “caret” package was employed to randomly segregate participants into a training set for model development and a validation set for external validation, adhering to a theoretical ratio of 7:3 (25). This division ensured that the model was developed on one subset of the data and validated on a separate, unseen subset.

This research used the “createDataPartition” function in the R language's “caret” package, aiming to facilitate stratified random sampling of the original dataset. The randomness principle of the “createDataPartition” function was based on stratified random sampling, a statistical technique where the entire population was divided into non-overlapping subgroups (e.g., whether a diagnosis of hypertension had been made), and then samples were drawn randomly from each subgroup. To achieve a training and validation set ratio of 7:3, indicating that 70% of the samples were allocated to the training set, the “createDataPartition” function extracted the requisite number of samples randomly within each stratum. Furthermore, the randomization process used by the “createDataPartition” function was based on a uniform distribution, suggesting that the probability of each sample being drawn was identical. To ensure the reproducibility of the results, a random seed was set using the “set.seed” function, guaranteeing consistency in the sampling results each time the code was executed. In conclusion, this methodology provided a 7:3 ratio of the number of samples in the training and validation sets, with no significant differences in the incidence of hypertension.

To determine the odds ratio of hypertension incidence, continuous variables were transformed into their categorical equivalents. The criteria for such categorization can be found in the original data, available in the Supplementary Files. The study prioritized the preservation of data integrity and precision.

A stringent complete-case analysis strategy was employed, which involved analyzing only those observations with complete data for all variables under consideration, thereby excluding any observation with even one missing value (26). This approach ensured the study's quality, even if it meant a potential reduction in the sample size. Moreover, this approach was chosen to sidestep assumptions that might distort research outcomes, ensuring the study's caliber, albeit with a potential reduction in sample size.

The LASSO regression algorithm, facilitated by the “glmnet” package, was applied exclusively to the entirety of the training set. This method, known for its prowess in shrinkage and variable selection for linear regression models, was designed to identify a subset of predictors by minimizing prediction error. It achieved this by imposing constraints on the model parameters, causing the regression coefficients for certain variables to shrink toward zero. Consequently, variables with coefficients that shrank to zero were deemed redundant and excluded. In contrast, variables with non-zero regression coefficients were identified as having a significant association with the dependent variable (27–29). It's worth noting that while BMI is mathematically derived from body weight and height, each of these metrics can represent distinct biological or epidemiological risk factors. By including all these variables in the LASSO regression, the study aimed to capture the multifaceted nature of potential predictors, allowing the algorithm to select the most pertinent ones, even if they exhibit collinearity.

The parameter “hypertension” was set as a binary variable because the included dependent variable confirmed if the participant could be diagnosed with hypertension. Based on the type of “2 log likelihood” and the binomial family, the LASSO regression analysis run in R software used a k-fold (10-fold in this study) cross-validation for centralization and normalization of the included variables and screened out the best lambda value. The “Lambda.lse” in the results of LASSO regression showed a model with good performance and the fewest number of independent variables. The effect sizes of these variables were the odds ratios with their *P*-value at all two-sided 95% confidence levels; the variables that had statistical significance were screened out and used to develop the nomogram predictive models. Then, a multivariable logistic regression analysis was used to construct the predictive models by introducing the features selected in the LASSO regression model with the help of the “rms” package (30).

To assess the accuracy of the risk nomograms, several validation techniques were applied, utilizing data from both the training and validation sets. Initially, the “pROC” package was used to compute the area under the receiver characteristic curve (AUC), indicating the specificity, sensitivity, and discrimination of the risk nomogram (31). Subsequently, calibration curves, computed using the “rms” package, were accompanied by the Hosmer–Lemeshow test to gauge the risk nomogram's calibration. Lastly, the decision curve analysis (DCA) was performed using the “nricens” package, determining the clinical applicability of nomograms based on the net benefit under varying threshold probabilities in the hypertension cohort (32). Both the Hosmer–Lemeshow test and the DCA iterations were set at 500 with 10-fold cross-validations.

Lastly, the clinical impact curves (CIC) and the net reduction curves plots (NRC) were outputted to evaluate the clinical applicability of the risk prediction nomogram by visually showing whether the nomogram possessed significant predictive value and had a superior overall net benefit within the wide and practical ranges of threshold probabilities and impacted participants' outcomes (33, 34).

## 3. Results

### 3.1. Participants

Data were meticulously collated from 24,709 adults who had partaken in the annual health assessment. Of this assembly, 3,452 individuals (13.97%) were excluded due to the absence of specific test results. A further 1,251 participants (5.06%) were omitted on account of aberrant data, identified as outliers. Additionally, 1,639 participants (6.63%) were excluded owing to the non-availability of blood samples. After these comprehensive exclusions, which encompassed 6,342 participants (a loss rate of 26.03%), the resultant dataset for rigorous statistical evaluation comprised 18,367 individuals.

It is imperative to highlight that the exclusions, especially those related to outliers, were carried out with rigorous precision to safeguard the integrity and validity of the findings. Outliers, by their very nature, can distort results and produce misleading interpretations. The removal of these outliers, as detailed in the methodology, ensures statistical accuracy, enhanced model performance, preservation of data normality, reduced variance, and the validity of the results. Meticulous measures were taken to ensure that the exclusion of these data points did not introduce discernible bias to the study.

**Figure 1:**
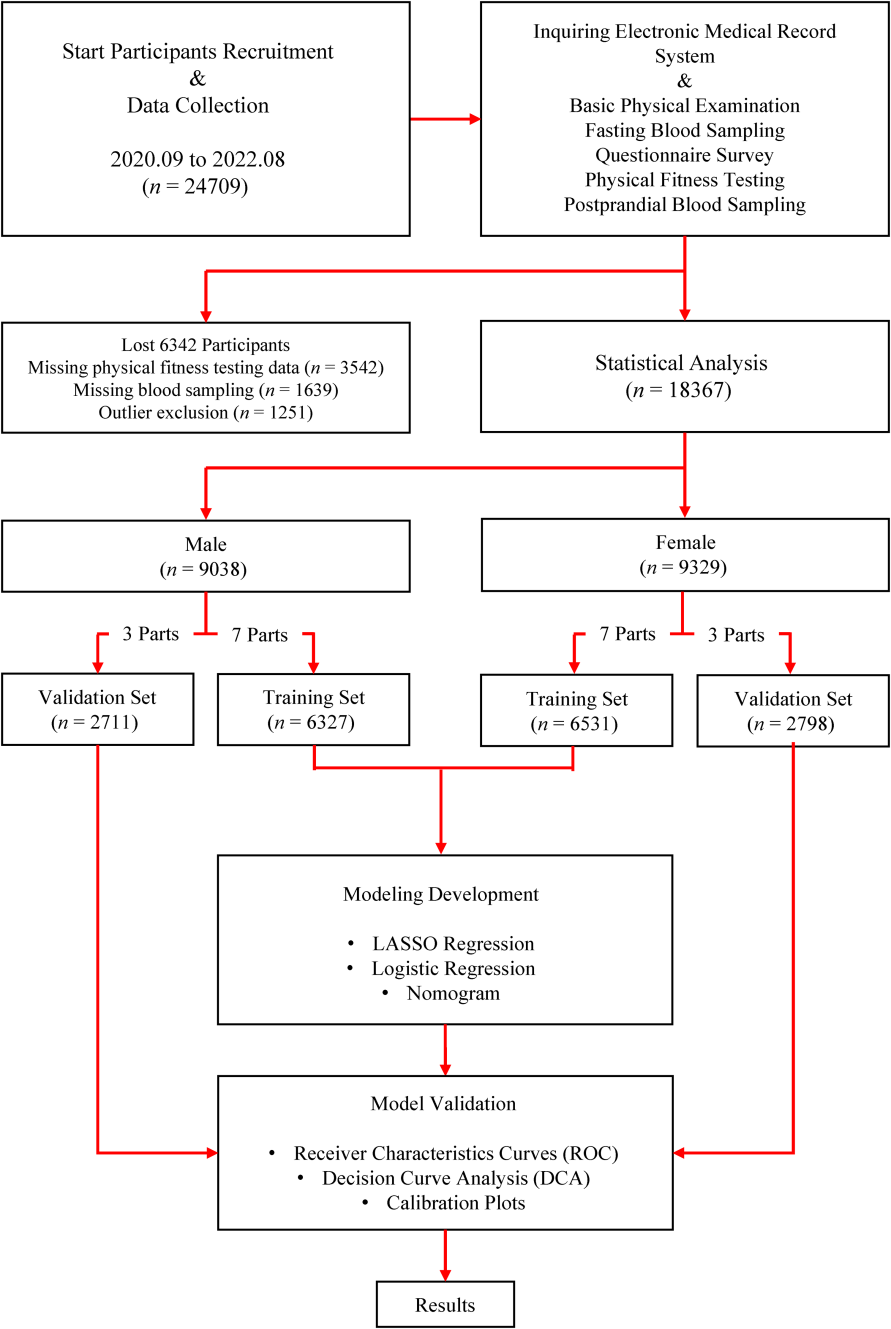
Flow diagram outlining the study process.

A total of 18,367 participants, encompassing both male and female individuals, were enrolled over the three years. These participants were subsequently and randomly allocated into a training set and a validation set, adhering to a theoretical ratio of 7:3. This bifurcation was essential for external validation. Figure 1 delineates the study's flow diagram, while Table 2 elucidates the characteristics of the participants, presented both collectively and separately by gender, to offer a comprehensive understanding of the study's demographic composition.

**Table 2:** Characteristics of the participants enrolled in the study according to the presence/absence of hypertension and randomly allocated into a training set and validation set.

Variables	Total (*n* = 18,367)	Hypertension (*n* = 9,343)	Non-Hypertension (*n* = 9,024)	Male			Female		
				Training set (*n* = 6,327)	Validation set (*n* = 2,711)	*P*-value	Training set (*n* = 6,531)	Validation set (*n* = 2,798)	*P*-value
Gender, No. (%) with data									
Male	9,038 (49.2)	5,558 (59.5)	3,480 (38.6)						
Female	9,329 (50.8)	3,785 (40.5)	5,544 (61.4)						
Age, Mean (95% CI), y	39.5 (29.9, 49.2)	43.6 (33.7, 52.5)	35.4 (27.5, 45.2)	39.8 (30.1, 49.9)	39.5 (29.3, 49.9)	0.495	39.4 (30.0, 48.6)	39.1 (29.9, 48.6)	0.549
Height, Mean (95% CI), cm	164 (158, 170)	166 (159, 171)	162 (157, 169)	170 (166, 174)	170 (166, 174)	0.200	159 (155, 162)	158 (155, 162)	0.608
Bodyweight, Mean (95% CI), kg	63.2 (56.0, 71.7)	67.5 (60.0, 75.0)	59.5 (53.2, 67.0)	70.0 (63.7, 77.2)	70.0 (63.9, 77.3)	0.742	57.3 (52.1, 63.0)	57.3 (52.2, 63.1)	0.387
BMI, Mean (95% CI), kg/mP^2^	23.5 (21.4, 25.8)	24.6 (22.5, 26.8)	22.5 (20.6, 24.6)	24.3 (22.3, 26.4)	24.3 (22.3, 26.4)	0.750	22.7 (20.8, 25.0)	22.8 (20.9, 25.1)	0.329
Weight Status, No. (%) with data						0.459			0.637
Underweight	592 (3.22)	145 (1.55)	447 (4.95)	113 (1.79)	61 (2.25)		286 (4.38)	132 (4.72)	
Normal	9,579 (52.2)	3,846 (41.2)	5,733 (63.5)	2,777 (43.9)	1,189 (43.9)		3,946 (60.4)	1,667 (59.6)	
Overweight	6,305 (34.3)	3,900 (41.7)	2,405 (26.7)	2,624 (41.5)	1,103 (40.7)		1,807 (27.7)	771 (27.6)	
Obesity	1,891 (10.3)	1,452 (15.5)	439 (4.86)	813 (12.8)	358 (13.2)		492 (7.53)	228 (8.15)	
Junk food, Mean (95% CI), times/week	2.00 (1.00, 3.00)	2.00 (1.00, 3.00)	2.00 (1.00, 3.00)	2.00 (1.00, 3.00)	2.00 (1.00, 3.00)	0.737	2.00 (1.00, 3.00)	2.00 (1.00, 3.00)	0.701
Sleeping, Mean (95% CI), hours/day	8.00 (7.00, 8.00)	8.00 (7.00, 8.00)	8.00 (7.00, 8.00)	8.00 (7.00, 8.00)	8.00 (7.00, 8.00)	0.516	8.00 (7.00, 8.00)	8.00 (7.00, 8.00)	0.463
Insomnia, No. (%) with data						0.504			0.390
Never	2,736 (14.9)	1,344 (14.4)	1,392 (15.4)	1,078 (17.0)	485 (17.9)		803 (12.3)	370 (13.2)	
Sometimes	10,242 (55.8)	5,047 (54.0)	5,195 (57.6)	3,640 (57.5)	1,527 (56.3)		3,576 (54.8)	1,499 (53.6)	
Often	5,389 (29.3)	2,952 (31.6)	2,437 (27.0)	1,609 (25.4)	699 (25.8)		2,152 (33.0)	929 (33.2)	
Daily activity level, No. (%) with data						0.631			0.133
High	2,611 (14.2)	1,263 (13.5)	1,348 (14.9)	1,144 (18.1)	470 (17.3)		711 (10.9)	286 (10.2)	
Moderate	6,397 (34.8)	3,051 (32.7)	3,346 (37.1)	2,238 (35.4)	955 (35.2)		2,274 (34.8)	930 (33.2)	
Low	9,359 (51.0)	5,029 (53.8)	4,330 (48.0)	2,945 (46.5)	1,286 (47.4)		3,546 (54.3)	1,582 (56.5)	
Chest circumference, Mean (95% CI), cm	89.0 (83.5, 94.0)	91.0 (86.0, 97.0)	86.0 (82.0, 91.0)	91.8 (87.0, 97.0)	92.0 (87.0, 97.0)	0.367	86.0 (81.0, 91.0)	86.0 (81.0, 91.0)	0.787
Waistline, Mean (95% CI), cm	80.0 (73.0, 88.0)	84.0 (77.0, 91.0)	76.0 (70.5, 83.0)	85.0 (78.2, 91.0)	85.0 (79.0, 91.5)	0.414	76.0 (70.0, 82.0)	76.0 (70.0, 82.5)	0.447
Hipline, Mean (95% CI), cm	93.5 (89.0, 98.0)	95.0 (91.0, 100)	92.0 (88.0, 96.0)	95.5 (91.0, 100)	95.7 (91.0, 100)	0.188	92.0 (88.0, 96.0)	92.0 (88.0, 96.0)	0.745
CVD History, No. (%) with data						0.024			0.466
No	15.0 (10.0, 20.0)	15.0 (10.0, 21.0)	15.0 (10.0, 20.0)	5,535 (87.5)	2,418 (89.2)		6,063 (92.8)	2,610 (93.3)	
Yes	18.0 (13.0, 23.0)	20.0 (15.0, 25.0)	17.0 (12.0, 22.0)	792 (12.5)	293 (10.8)		468 (7.17)	188 (6.72)	
RHR, Mean (95% CI), beats/min	76.0 (71.0, 83.0)	78.0 (72.0, 84.0)	75.0 (70.0, 82.0)	76.0 (71.0, 83.0)	76.0 (71.0, 83.5)	0.791	76.0 (71.0, 83.0)	76.0 (72.0, 83.0)	0.289
FVC, Mean (95% CI), ml	2,870 (2,210, 3,596)	2,986 (2,244, 3,696)	2,760 (2,185, 3,458)	3,553 (3,028, 4,094)	3,568 (3,017, 4,110)	0.820	2,330 (1,896, 2,764)	2,300 (1,900, 2,730)	0.259
Grip strength, Mean (95% CI), kg	35.0 (27.3, 46.3)	39.6 (29.1, 48.3)	31.6 (26.1, 43.1)	46.2 (40.9, 51.4)	46.5 (41.4, 51.4)	0.328	27.7 (24.1, 31.3)	27.7 (24.1, 31.4)	0.922
Grip/BW, Mean (95% CI), kg/kg	0.56 (0.47, 0.67)	0.57 (0.47, 0.67)	0.55 (0.46, 0.66)	0.66 (0.58, 0.74)	0.66 (0.58, 0.74)	0.913	0.48 (0.42, 0.55)	0.48 (0.42, 0.55)	0.395
Grip/BMI, Mean (95% CI), mP^2^	1.51 (1.19, 1.91)	1.59 (1.21, 1.94)	1.44 (1.17, 1.86)	1.90 (1.66, 2.15)	1.90 (1.66, 2.16)	0.475	1.22 (1.04, 1.40)	1.21 (1.03, 1.40)	0.325

BMI, body mass index; CVD, cardiovascular diseases; RHR, resting heart rate; FVC, forced vital capacity; Grip/BW, ratio of grip strength and body weight; Grip/BMI, ratio of grip strength and body mass index.

### 3.2. Model development

The results of the logistic regression analysis in the training sets are listed in Table 3. From the results of the multivariate logistic regression analysis in the training sets, it can be observed that for the risk prediction of hypertension in men, there are 5 independent predictors: age, BMI, waistline, hipline, and resting heart rate identified in the risk predictive model. Meanwhile, for the prediction of hypertension in women, 7 independent predictors are identified: age, BMI, body weight, CVD history, waistline, resting heart rate, and daily activity level (DAL). The practical implications of these predictors are manifold. For instance, the identified predictors can be used by healthcare professionals to develop personalized risk assessment tools. These tools can aid in early identification of individuals at risk, allowing for timely interventions. Moreover, public health campaigns can focus on these predictors, emphasizing the importance of maintaining a healthy BMI, waistline, and regular monitoring of resting heart rate. The inclusion of factors such as CVD history and DAL further underscores the need for comprehensive health assessments, integrating both medical history and lifestyle factors. In essence, these predictors can serve as a foundation for holistic health strategies aimed at reducing the prevalence of hypertension in the community.

**Table 3:** Logistic regression analysis of the predictors for the risk of hypertension.

Gender	Variables	Estimate (B)	*z* value	In predictive model				
				Estimate (B)	*z* value	Odds ratio	95% CI	*P*-value
Male	RHR	0.205	9.312	0.226	9.717	1.254	1.198–1.312	<0.001
	Hipline	0.549	14.73	0.154	3.042	1.166	1.056–1.288	0.002
	Waistline	0.544	18.682	0.134	2.973	1.143	1.047–1.249	0.003
	BMI	0.182	19.539	0.114	8.527	1.121	1.092–1.15	<0.001
	Age	0.355	14.872	0.312	12.23	1.366	1.3–1.436	<0.001
Female	DAL	0.349	9.078	0.214	5.107	1.238	1.141–1.344	<0.001
	RHR	0.134	6.321	0.236	10.042	1.266	1.209–1.325	<0.001
	Waistline	0.644	21.661	0.257	5.887	1.293	1.187–1.409	<0.001
	CVD History	0.844	8.637	0.367	3.465	1.443	1.173–1.776	0.001
	BMI	0.184	21.022	0.059	3.458	1.06	1.026–1.096	0.001
	Bodyweight	0.578	18.77	0.113	2.018	1.12	1.003–1.25	0.044
	Age	0.544	22.064	0.409	14.588	1.505	1.425–1.59	<0.001

RHR, resting heart rate; BMI, body mass index; DAL, daily activity level; CVD, cardiovascular diseases.

**Figure 2:**
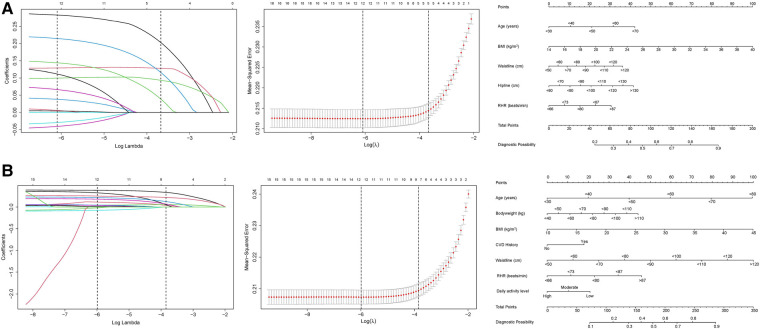
Variable selection by the Least absolute shrinkage and selection operator (LASSO) binary logistic regression models and the corresponding nomograms. The left plots are the coefficient profile plots that are constructed against the log(lambda) sequence to show the selection process with variables selection process with nonzero coefficients by deriving the optimal lambda for the models. The middle plots are the dotted vertical lines at the optimal values by using the 1 standard error of the minimum criteria (lambda.1se). The right plots are nomogram predictive models of risk factors selected. (**A**) Male; (**B**) Female.

Figure 2 outlines the process of variable selection by the LASSO binary logistic regression models and the nomograms of the independent hypertension predictors. In the nomograms, the categorical variables are displayed as their cut-off points. The nomograms can also represent the structures of the models and be used to calculate the probability of hypertension incidence. According to the value of each predictor in the nomogram, the corresponding score can be obtained on the number line of the first row and added sequentially. The total score calculated can then be used to determine the probability of the disease on the number line representing the probability of diagnosis in the last row.

### 3.3. Model validation

**Figure 3:**
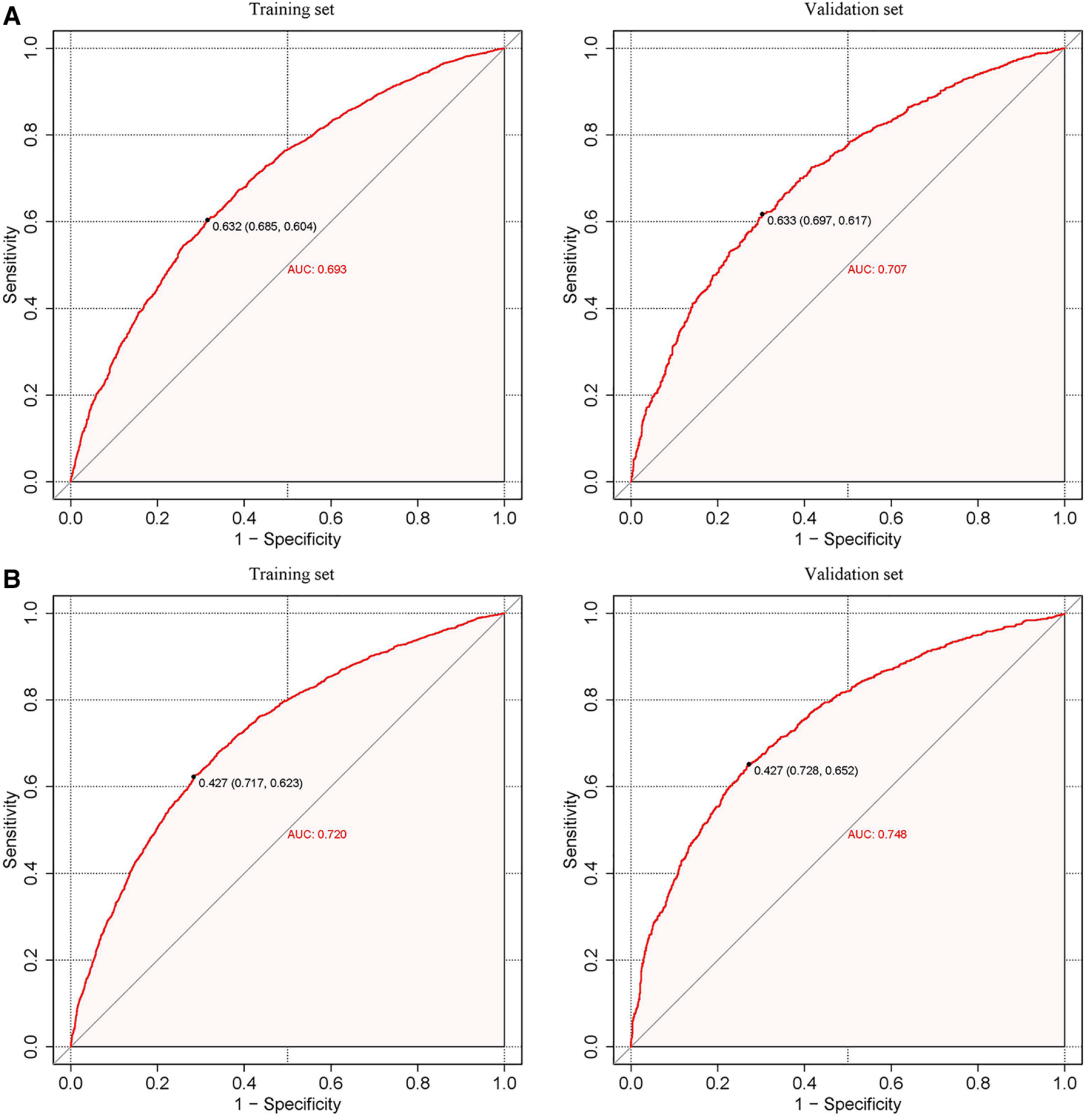
Calibration curves of the predictive hypertension risk nomograms. The y-axis represents actual diagnosed cases of hypertension, while the x-axis represents the predicted risk of hypertension. The diagonal dotted lines represent a perfect prediction by an ideal model, and the solid blue line and red line represent the performance of the training set (left) and validation set (right) before and after calibration. A closer fit between the solid and diagonal dotted lines indicates a better prediction performance. (**A**) Male; (**B**) Female.

Figure 3 displays the receiver operating characteristic curves (ROC) of the nomograms, which show their discriminations, specificities, and sensitivities. As per Figure 3, for the male hypertension risk predictive nomogram, the pooled area under the ROCs (AUC), representing the model's discrimination, is 0.693 in the training set and 0.707 in the validation set. For the female hypertension risk predictive nomogram, the AUC is 0.720 in the training set and 0.748 in the validation set. To contextualize these values, an AUC value between 0.7 and 0.8 is generally considered acceptable, and our results fall within this range. When benchmarked against other studies, our nomograms' performance is consistent with several other predictive models for hypertension, further validating the reliability of our findings. It's worth noting that while our nomograms demonstrate moderate performance, they are based on easily measurable parameters, making them practical for real-world applications.

**Figure 4:**
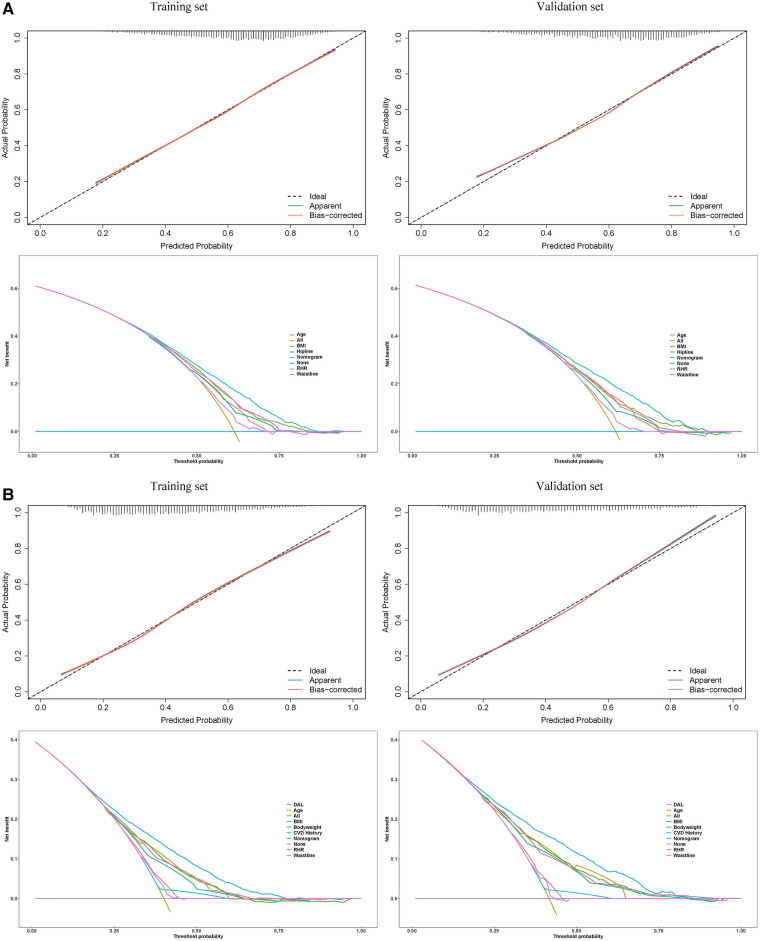
Calibration curves (upper) of the predictive hypertension risk nomograms and the decision curve analysis (lower) for the hypertension risk nomograms. In the calibration curves, the y-axis represents actual diagnosed cases of hypertension, while the x-axis represents the predicted risk of hypertension. The diagonal dotted lines represent a perfect prediction by an ideal model, and the solid blue line and red line represent the performance of the training set and validation set before and after calibration. A closer fit between the solid and diagonal dotted lines indicates a better prediction performance. In the decision curve analysis, the y-axis measures the net benefit. The horizontal lines named “None” represents the assumption that no participant had hypertension. The lines named “All” represents the assumption that all participants have hypertension, the lines named “nomogram” represents the predictive model established, and other lines represent the risk models of the risk factors they refer to. The left plots are from the training sets, and the right plots are from the validation sets. (**A**) Male; (**B**) Female.

**Figure 5:**
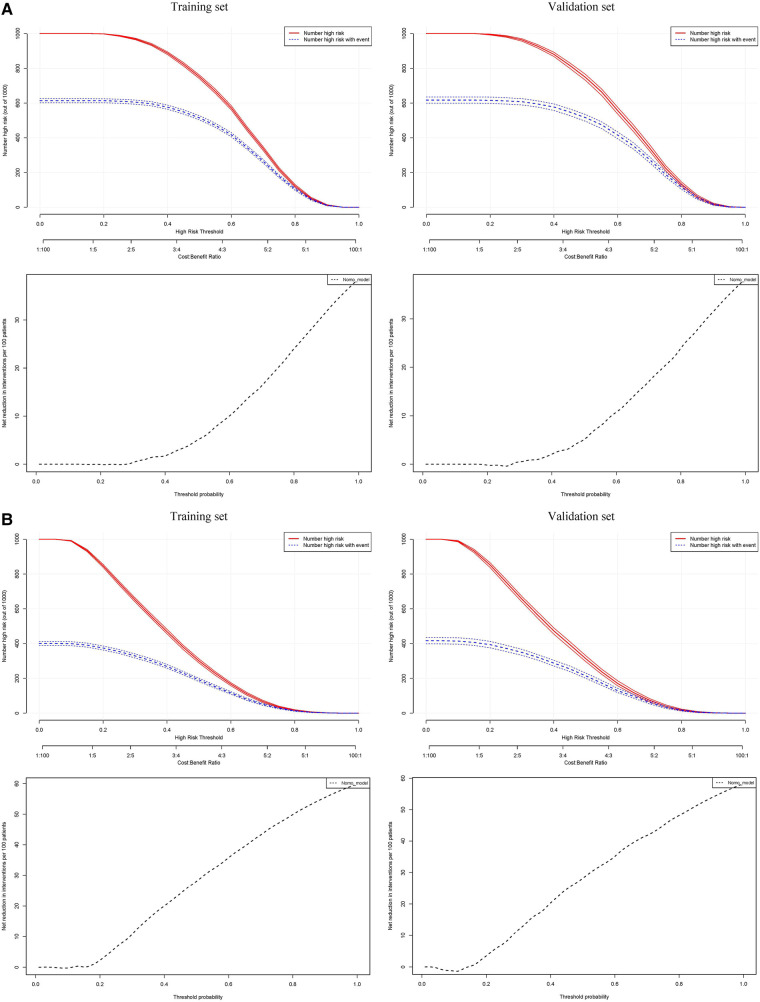
In the clinical impact curves (CIC) of the nomograms (upper) and the net reduction curves (NRC) of the nomograms (lower). In the CICs, the red curve (number of high-risk individuals) indicates the number of people who are classified as positive (high risk) by the model at each threshold probability; the blue curve (number of high-risk individuals with the outcome) is the number of true positives at each threshold probability. In the NRCs, the values on the y-axis represent the number of patients that could be reduced under the same effect size by using a certain threshold probability of diagnosis (the value on the x-axis). The left plots are from the training sets, and the right plots are from the validation sets. (**A**) Male; (**B**) Female.

Figure 4 presents the calibration plots based on the Hosmer–Lemeshow tests and the results of the decision curve analysis (DCA), which display the threshold probabilities of the predictive nomograms. Figure 5 provides the clinical impact curves (CIC) and the net reduction curve (NRC) plots of the nomograms in the training and validation sets.

The calibration curves in the hypertension predictive nomograms for both men and women reveal that the predictive performance in the training sets and the validation sets are well-fitted. The net benefit in the decision curves of all the nomograms is greater than any model established by a single factor. Furthermore, the CICs in the training and validation sets and the NRC plots are also well-fitted. The consistency between the models in the training set and validation set from various perspectives indicates that the nomograms possess high net benefits (predictive power) in clinical practice.

## 4. Discussion

Based on the risk-predictive nomograms for hypertension established for male and female participants, three salient findings emerged from this study. Firstly, while age was a non-modifiable risk factor, BMI, waistline, and RHR were modifiable risk factors for hypertension in both males and females. Secondly, while our predictive nomograms highlighted gender differences, it is crucial to note that both genders exhibit certain commonalities in hypertension risk prediction. Indeed, a history of CVD and DAL is recognized as significant risk predictors of hypertension, not just in the Chinese female population but also in males. However, our study aimed to underscore the unique predictors that might introduce heterogeneity in hypertension risk prediction between the two groups. Lastly, beyond basic demographic and anthropometric parameters such as age, body weight, and trunk circumferences, information about the functional status of the cardiovascular system and physical activity was pivotal in predicting the risk of hypertension.

The findings of this study harmonize with a rich tapestry of research that underscores age, BMI, and waistline as pivotal predictors of hypertension. For instance, a study published in 2017 delineated a robust association between advancing age and the susceptibility to hypertension, mirroring our discernments (35). The accentuation on malleable risk determinants such as BMI and waistline finds consonance with a study published in 2015, which illuminated the transformative potential of lifestyle recalibrations in circumscribing hypertension (36). The gender-centric predictors we spotlighted find parallels in the revelations of, insinuating that the gender-driven variances in hypertension risk transcend regional confines and bear global ramifications (37). The salience of functional status and physical activity in our exploration, while corroborated by extant literature, accentuates the cardinality of cardiovascular well-being and quotidian activity cadence in the genesis and evolution of hypertension. In essence, our exposition, while unveiling avant-garde insights, is anchored in the expansive saga of hypertension research, forging connections and proffering novel vantage points.

Since the variables of age, BMI, waistline, and RHR were included in both the hypertension risk nomograms for men and women, it indicated that these variables might be the generic predictors for hypertension in Chinese adults. This inference had been supported by many previous studies. For example, in 2018, Kjeldsen published a review of hypertension and cardiovascular risk, claiming that hypertension, whose overall prevalence increased steeply with aging, was one of the strongest risk factors for almost all different cardiovascular diseases acquired during life (38). Further to this, in a Chinese population, a study by Du's team demonstrated that the increased risk of self-reported hypertension prevalence was associated with age, marital status, drinking, BMI, and comorbidity (39). About the waistline, Cho et al. suggested that waistline had a better predictive performance for hypertension than BMI, age, and gender in an Asian population and was a more sensitive marker of hypertension in younger people than in the elderly (40). RHR, which was one of the parameters that represented the function of the cardiovascular system, had been verified to have a positive correlation with the risk of hypertension, and individuals with normal blood pressure could be at an increased risk for future hypertension if the ability of cardio autonomic control was reduced (41–43).

Nevertheless, the predictors and their weight coefficients in the risk nomograms of hypertension for Chinese male and female adults were different. The heterogeneity might have been induced by the gender difference with potential mechanisms emanating from the following perspectives. On one hand, gender differences existed in some risk factors directly related to hypertension, such as waistline and hipline (44–46), which might have led to different weight coefficients for these factors in hypertension risk prediction models for men and women. On the other hand, the effects of some mediating-moderating factors on hypertension risk were different between genders (47, 48). For example, a higher muscular strength appeared to be associated with a lower incidence of hypertension (49, 50), however, the muscle strength of men was generally higher than that of women. At the same time, the loss of muscle volume and the rate of muscle strength decline in women were higher than those in men (51).

Finally, the construction of risk predictors seemed to indicate that to predict the risk of hypertension, information from three perspectives was required. Except for basic demographic and anthropometric parameters such as age, body weight, body height, and trunk circumferences, to predict the risk of hypertension, information about the functional status of the cardiovascular system and physical activity seemed necessary. According to the results of this study, heart rate, which was linked to blood pressure, represented the fact that cardio function directly affected blood pressure. A lower heart rate represented a higher stroke volume and a higher ejection fraction, indicating better cardiovascular system function and a lower risk of hypertension (52–56). Moreover, the history of CVD could directly represent the function of the cardiovascular system. For example, individuals with hyperglycemia or/and hyperlipidemia had a higher risk of hypertension (57–59), and physical activity, especially aerobic exercise, could improve the function of the cardiovascular system and/or muscle strength, which were negatively correlated with hypertension (60, 61). Future studies should focus on the predictive power of other indicators of cardiovascular function and physical fitness to optimize hypertension risk prediction models and improve their predictive performance.

The study, while being rigorous, has certain limitations. The age distribution of the participants skewed, with a notable lack of representation from males aged over 70. As a result, grip strength may not comprehensively reflect whole-body strength. Moreover, the elevated prevalence of hypertension observed in this study can be attributed to the higher average age of the participants, which inherently predisposes them to a greater risk of hypertension. A pivotal limitation to note is the exclusion of baseline blood pressure from our analysis. While its diagnostic value is undeniable, we aimed to emphasize other easily measurable parameters for hypertension prediction. Another significant limitation is the omission of well-established factors related to hypertension, such as smoking status, blood glucose, and lipids. Their inclusion might have provided a more holistic view of hypertension risk. It's also worth noting that the risk predictors were identified using a cross-sectional design. A longitudinal design would have been more accurate in this context. Although all blood samples were meticulously collected on-site, the family history of hypertension or other cardiovascular diseases wasn’t investigated. This oversight prevents an analysis of genetic predispositions toward hypertension (62, 63). A significant point to consider is that the definition of hypertension was based on standards prevailing during the participant inclusion period (2020–2022), and not the latest 2023 guidelines. This might have implications on the study outcomes and interpretations. Furthermore, the methodology used to segment continuous variables, such as age, BMI, hipline, waistline, and grip strength, relied on linear regression. Future studies should consider using optimal scaling regression to identify more nuanced cut-off values for the categorization of these continuous variables (64, 65). Additionally, while transforming continuous predictors into categorical ones can be advantageous in certain contexts, it may result in a potential loss of information. The selection of boundaries for categorization can be subjective, potentially influencing results, and having too many categories could unintentionally make the model more complex. Notably, while the nomograms demonstrated moderate performance, they may not rival the predictive prowess of some existing models. This moderate performance, despite the simplicity of parameters, underscores the intricate nature of hypertension prediction and the potential influence of unmeasured confounders.

## 5. Conclusion

Gender differences may introduce heterogeneity in hypertension risk prediction between men and women. In predicting hypertension risk for the female population, besides basic demographic and anthropometric parameters, information regarding the functional status of the cardiovascular system and physical activity also seems necessary. Furthermore, the insights gleaned from our study hold profound implications for healthcare practitioners. By understanding these gender-specific predictors, clinicians can tailor preventive strategies and interventions more effectively. This personalized approach not only aids in early detection but also fosters a proactive healthcare model, emphasizing prevention over cure. In essence, our findings serve as a beacon, guiding healthcare professionals in their quest to mitigate the burgeoning menace of hypertension in diverse populations.

## Data Availability

The datasets used in this study can be found in the article/supplementary material. Further enquiries can be directed to the corresponding author(s).
